# Population Pharmacokinetic Modeling of Total and Unbound Pamiparib in Glioblastoma Patients: Insights into Drug Disposition and Dosing Optimization

**DOI:** 10.3390/pharmaceutics17040524

**Published:** 2025-04-16

**Authors:** Charuka Wickramasinghe, Seongho Kim, Yuanyuan Jiang, Xun Bao, Yang Yue, Jun Jiang, Amy Hong, Nader Sanai, Jing Li

**Affiliations:** 1 Karmanos Cancer Institute, Wayne State University School of Medicine, Detroit, MI 48201, USA; 2 Barrow Neurological Institute, St. Joseph’s Hospital & Medical Center, Phoenix, AZ 85013, USA

**Keywords:** population pharmacokinetic model, pamiparib, PARP inhibitor, unbound plasma concentrations, dosing optimization, glioblastoma

## Abstract

**Background:** This study aimed to develop a population pharmacokinetic (PK) model that characterized the plasma concentration–time profiles of the total and unbound pamiparib, a PARP inhibitor, in glioblastoma patients and identified patient factors influencing the PK. **Methods:** The total and unbound pamiparib plasma concentration data were obtained from 41 glioblastoma patients receiving 60 mg of pamiparib twice daily. Nonlinear mixed-effects modeling was performed using Monolix (2024R1) to simultaneously fit the total and unbound drug plasma concentration data. The covariate model was developed by covariate screening using generalized additive modeling followed by stepwise covariate modeling. Model simulations were performed following oral doses of 10–60 mg BID. **Results:** The total and unbound pamiparib plasma concentration–time profiles were best described by a one-compartment model with first-order absorption and elimination. Creatinine clearance and age were the significant covariates on the apparent volume of distribution (V/F) and apparent clearance (CL/F), respectively, explaining ~22% and ~5% of IIV of V/F and CL/F. Population estimates of the absorption rate constant (Ka), V/F, CL/F, and unbound fraction for the total drug were 1.58 h^−1^, 44 L, 2.59 L/h, and 0.041. Model simulations suggested that doses as low as 20 mg BID may be adequate for therapeutic effects in a general patient population, assuming that a target engagement ratio (i.e., unbound C_ss,min_/IC50) of 5 or above is sufficient for full target engagement. **Conclusions:** The total and unbound pamiparib plasma PK are well characterized by a linear one-compartment model, with creatinine clearance as the significant covariate on V/F. Model simulations support further clinical investigation into dose reduction to optimize the benefit-to-risk ratio of pamiparib, particularly in combination therapies.

## 1. Introduction

Pamiparib is a selective, orally bioavailable inhibitor of Poly (ADP-ribose) polymerase-1 (PARP1) and PARP2, enzymes that play a crucial role in DNA damage repair [1,2]. PARP enzymes detect and repair single-strand DNA breaks (SSBs) through the base excision repair (BER) pathway. Pamiparib binds to the catalytic domain of PARP1/2, preventing them from synthesizing Poly (ADP-ribose) (PAR) chains, which are essential for recruiting DNA repair proteins [1,2]. In addition, pamiparib traps PARP1/2 on damaged DNA, leading to the accumulation of double-strand breaks, which are particularly lethal to cancer cells with defective homologous recombination (HR) repair mechanisms [1]. Tumors with mutations in BRCA1 or BRCA2 (key HR repair genes) are unable to repair double-strand breaks effectively. As such, pamiparib exploits synthetic lethality to selectively target cancer cells with DNA repair deficiencies while sparing normal cells, making it a potential therapeutic option for tumors with BRCA mutations and other DNA damage repair defects [1,3].

Pamiparib has been extensively evaluated in clinical trials for treating a variety of cancers, particularly those associated with DNA damage repair deficiencies such as BRCA-mutated malignancies. A phase I study demonstrated that pamiparib was well tolerated and exhibited preliminary antitumor activity in patients with high-grade epithelial non-mucinous ovarian cancer [4]. A phase II trial demonstrated the significant clinical benefit of pamiparib [3], leading to its approval in China for the treatment of germline BRCA mutation-associated recurrent advanced ovarian, fallopian tube, or primary peritoneal cancer in patients previously treated with two or more lines of chemotherapy. In addition, pamiparib, as a monotherapy or in combination with chemotherapy or checkpoint inhibitors, has been evaluated in other solid tumors, including triple-negative breast cancer, prostate cancer, and advanced gastric cancer [5,6,7,8].

Notably, unlike some other PARP inhibitors, pamiparib is not a substrate of P-glycoprotein (P-gp), which enhances its ability to cross the blood–brain barrier (BBB), making it a promising candidate for treating brain tumors, such as glioblastoma [1,9]. In a phase 0/II trial, pamiparib demonstrated good central nervous system (CNS) penetration, achieving potentially therapeutic concentrations in both enhancing and non-enhancing tumors of glioblastoma patients [10]. Leveraging its ability to cross the BBB and potentiate DNA damage in cancer cells, pamiparib is under clinical evaluation in combination with temozolomide and radiation therapy for patients with newly diagnosed or recurrent gliomas.

The recommended dose of pamiparib is 60 mg twice daily, which is administered continuously until disease progression or unacceptable toxicity. However, due to significant interindividual pharmacokinetic (PK) variability, a one-size-fits-all dosing approach may lead to suboptimal treatment outcomes. Tailored dosing regimen for pamiparib is crucial to maximizing its therapeutic efficacy while minimizing toxicity in individual patients. Population pharmacokinetic (PK) modeling can aid in dose optimization by quantifying patient variability in drug disposition and identifying key factors influencing this process. This study aimed to develop a population PK model to characterize the plasma concentration–time profiles of total and unbound pamiparib in glioblastoma patients and to identify patient characteristics (such as demographics, renal and hepatic function, and combination therapy) that contribute to PK variability. The findings provided critical insights into the drug disposition and have clinical implications for guiding the optimization of dosing strategies in patients with glioblastoma and other cancers.

## 2. Materials and Methods

### 2.1. Clinical Study and PK Data

The total and unbound pamiparib plasma concentration data used in the population PK analysis were obtained from a phase 0/II study (ClinicalTrials.gov ID: NCT04614909), which enrolled 41 patients with newly diagnosed or recurrent glioblastoma. The protocols were approved by the Institutional Review Board of the Barrow Neurological Institute. Written informed consent was obtained from all participants.

Eligible patients were required to have newly diagnosed or recurrent glioblastoma and meet the following criteria: age >18 years, Eastern Cooperative Oncology Group (ECOG) performance status ≤2, and adequate bone marrow and organ functions. Adequate bone marrow function was defined as an absolute neutrophil count ≥1.5 × 10⁹/L, a platelet count ≥100 × 10⁹/L, and hemoglobin ≥ 9 g/dL. Adequate liver function was defined as serum transaminase (AST and ALT) levels ≤ 3 times the institutional upper limit of normal (ULN) and total bilirubin ≤ 1.5 × ULN. Adequate kidney function was defined as a glomerular filtration rate (eGFR) ≥ 30 mL/min/1.73 m^2^ estimated by the Chronic Disease Epidemiology Collaboration (CKD-EPI) equation, and either serum creatinine ≤ 1.5 × ULN or creatinine clearance ≥60 mL/min for those with serum creatinine levels above 1.5 × ULN. Patients were required to have discontinued any prior anticancer treatment for at least 21 days before study entry. Patient demographic and characteristic data are summarized in Table 1.

The patients received oral pamiparib (60 mg twice daily) for three days before undergoing surgical tumor resection. Blood samples were collected at predose and at 0.5, 1, 2, 4, 7, and 24 h following the 9th oral dose. Plasma was separated from whole blood by centrifugation at 1000× *g* and 4 °C for 10 min and then stored at −80 °C until analysis. The total and unbound pamiparib concentrations in plasma samples were quantified using a validated liquid chromatography coupled with tandem mass spectrometry (LC-MS/MS) method [11].

### 2.2. Population PK Modeling Analysis

The population PK model was developed in two steps: (1) basic (structural) model development and (2) covariate model development. All analyses were performed using the nonlinear mixed-effects modeling program MONOLIX version 2024R1 (Lixoft, Antony, France).

#### 2.2.1. Structure Model Development

The structural model was developed to simultaneously characterize total and unbound pamiparib plasma concentration–time profiles following an oral administration of 9 doses (60 mg, twice daily) in individual glioblastoma patients. A one-compartment model with first-order absorption (without lag time) and first-order elimination was selected to describe the multiple-dose plasma concentration–time data. The primary PK parameters included apparent oral clearance (CL/F), apparent volume of distribution (V/F), absorption rate constant (Ka), and fraction of unbound drug in plasma (Fu). The relationship between total (Cp) and unbound (Cu) drug concentrations was incorporated into the model using the equation Cu = Cp × Fu. All concentration data were log-transformed to stabilize variance and improve model fitting. Population mean PK parameters, interindividual variability, and residual error were assessed in the population PK model. Interindividual variability was modeled using an exponential function, assuming log-normal distribution for all PK parameters.

Various residual error models, including constant, proportional, and combined error models, were evaluated. The combined model, incorporating both proportional and additive error components, was selected as the optimal error model (Equation (1)).

(1) $$y_{i,j}=f\left(t_{i,j},\psi_{i}\right)+g(t_{i,j},\psi_{i},\xi )\varepsilon_{i,j}$$

Here, $\psi_{i}$ is the parameter vector of the structural model f for individual i, and the residual error model is defined by the function g that depends on an additional parameter vector ξ. The residual errors $\varepsilon_{i,j}$ are standardized Gaussian random variables with a mean of 0 and a standard deviation of 1. In this analysis, the combined error model was defined as $g=\left(a+bf^{c}\right)$, where the function g consists of a constant term, a, and a term proportional to the structural model f, with b as the proportionality constant. The parameter vector ξ includes a and b, while c is fixed at 1 by default.

Population mean PK parameters were estimated using the Stochastic Approximation Expectation–Maximization (SAEM) algorithm, a robust method for handling complex nonlinear mixed-effects models. Individual patients’ PK parameter estimates were obtained through post hoc Bayesian estimation, utilizing the conditional distributions derived from the Markov Chain Monte Carlo (MCMC) procedure.

#### 2.2.2. Covariate Model Development

The covariate model was developed using a stepwise approach to systematically identify and incorporate significant covariate effects. Initial screening for potential covariates was conducted using generalized additive modeling (GAM) with R version 4.4.1. The candidate covariates, listed in Table 1, were evaluated based on their relationship with Bayesian-estimated individual pharmacokinetic (PK) parameters from the structural model, including CL/F, V/F, Ka, and Fu. For patients with missing covariate data, values were imputed using the population median, as the proportion of missing data was low (<2%). Continuous covariates (e.g., body surface area [BSA]) were centered around their median values, while categorical covariates were encoded as binary variables (e.g., male = 1 and female = 0). Potentially significant covariates identified via GAM were further assessed in the stepwise covariate modeling (SCM) procedure, where covariate relationships were explored using linear, exponential, or power functions. The exponential function (as shown in Equation (2)) was identified to best describe the covariate effects.

(2) $$\theta_{i}=\theta_{pop}\times e^{\beta_{COV}\times {COV}_{j}}$$

Here, $\theta_{i}$ represents the PK parameter for the $i^{th}$ patient, $\theta_{pop}$ is the mean population value of the parameter, $\beta_{COV}$ is the estimated covariate effect coefficient, and ${COV}_{j}$ is the identified covariate.

Discrimination between hierarchical models was based on both statistical and graphical criteria. First, model comparisons were conducted using the likelihood ratio test, where a reduction in OFV (−2 log-likelihood, −2LL) was used to assess model improvement. Covariates were retained if the addition of the covariate resulted in a reduction in OFV > 3.875 (*p* < 0.05) during forward selection and if the removal of the covariate led to an increase in OFV > 10.828 (*p* < 0.001) during backward elimination. In addition, model improvement was visually assessed through diagnostic goodness-of-fit plots, including observed vs. predicted concentrations to check for bias and weighted residuals vs. predicted concentrations to ensure random scatter around zero. Additional criteria for covariate inclusion were the increased precision in parameter estimates (i.e., reduced relative standard error [RSE]) and a reduction in interindividual variability of key PK parameters. Collectively, by integrating statistical selection criteria with graphical diagnostics, the final covariate model was refined to optimize predictive performance and biological plausibility.

### 2.3. Model Evaluation

The precision and stability of parameter estimates were assessed by bootstrap analysis with R (version 4.4.1) [12,13,14]. This nonparametric resampling method involves generating multiple datasets by randomly sampling, with replacement, from the original dataset. Each resampled dataset was used to re-estimate the model parameters, and this process was repeated 500 times to construct an empirical distribution of parameter estimates. The median and 95% confidence intervals (CIs) of the bootstrap-derived estimates were computed to evaluate the robustness of the parameter estimates. The agreement between the original parameter estimates and the mean of the bootstrap replicates was checked to confirm model stability and ensure that parameter uncertainty was adequately captured [12,13,14].

The predictive performance of the model was assessed by visual predictive checks (VPCs), which involve simulating 500 datasets using the estimated population parameters and then comparing the simulated concentration–time profiles to the observed data [15,16]. The 50th percentile (median) and the 5th and 95th percentiles (forming the 95% prediction interval) of the predicted concentrations were plotted alongside the observed concentrations. If most observed data points fall within the 95% prediction interval and align well with the simulated percentiles, it indicates that the model can adequately capture the central trend and variability in the data. A systematic deviation between observed and simulated percentiles may suggest model misspecification or the need for further refinement.

In addition, prediction distribution plots were constructed to compare observed concentration data with the theoretical distribution of model-predicted data [17]. Unlike standard VPCs, these plots rely on multiple simulations of the individuals in the dataset without residual error, focusing on the variability arising from structural and random-effects components alone [17]. These simulations use the estimated fixed and random effects from the population model while ignoring parameter uncertainty. By doing so, prediction distribution plots help identify potential systematic deviations between observed data and model predictions, particularly in the presence of model misspecification, overdispersion, or unexplained variability.

### 2.4. Model Simulations

To guide dose optimization, the developed population PK model was utilized to predict the unbound pamiparib plasma concentration–time profiles following twice-daily oral administration at doses of 10, 20, 30, 40, and 60 mg. Simulations of 200 datasets were performed using the estimated population parameters along with their respective interindividual variability. The median (50th percentile) and 5th and 95th percentiles of the predicted concentration–time profiles were generated. All simulations were executed using R (version 4.4.1).

## 3. Results

### 3.1. Population PK Model

The total and unbound pamiparib plasma concentration–time profiles following multiple-dose oral administration were well described by a one-compartment model with first-order absorption (with no lag time) and first-order elimination. Stepwise covariate model development identified creatinine clearance as a significant covariate on the V/F of both the total and unbound drug, explaining ~23% and ~22% of the respective IIV, and age as a significant covariate on the CL/F of the total and unbound drug, explaining ~ 6% and ~4% of the respective IIV. As such, the final covariate model consisted of a one-compartment model with first-order absorption and first-order elimination, incorporating creatine clearance as a covariate on pamiparib V/F and age as a covariate on the CL/F.

The population PK parameters for the total and unbound pamiparib along with their respective interindividual variability (IIV) estimates, from the structural and final covariate models, are summarized in Table 2. The population mean absorption rate constant (Ka) was 1.58 h⁻¹, with substantial IIV (~400%), indicating rapid but variable oral absorption. The population mean CL/F for total pamiparib was 2.59 L/h, with an IIV of 50%. The inclusion of age as a covariate on CL/F accounted for only 6% of the unexplained IIV, suggesting that other factors, which were not assessed in this study, may contribute more significantly to variability in pamiparib clearance. The estimated population mean V/F for total pamiparib was 44 L, suggesting a distribution volume exceeding blood volume. The addition of creatinine clearance as a covariate on V/F explained approximately 23% of the IIV. The population mean elimination half-life (T_1/2_) was estimated to be ~12 h, which is consistent with the estimated T_1/2_ of 13 h in the first in-human study (BGB-290-AU-002; NCT02361723) [4] and supporting a twice-daily dosing regimen. The population mean fraction of unbound pamiparib in plasma was 0.041, indicating high plasma protein binding.

### 3.2. Model Evaluation

**Figure 1:**
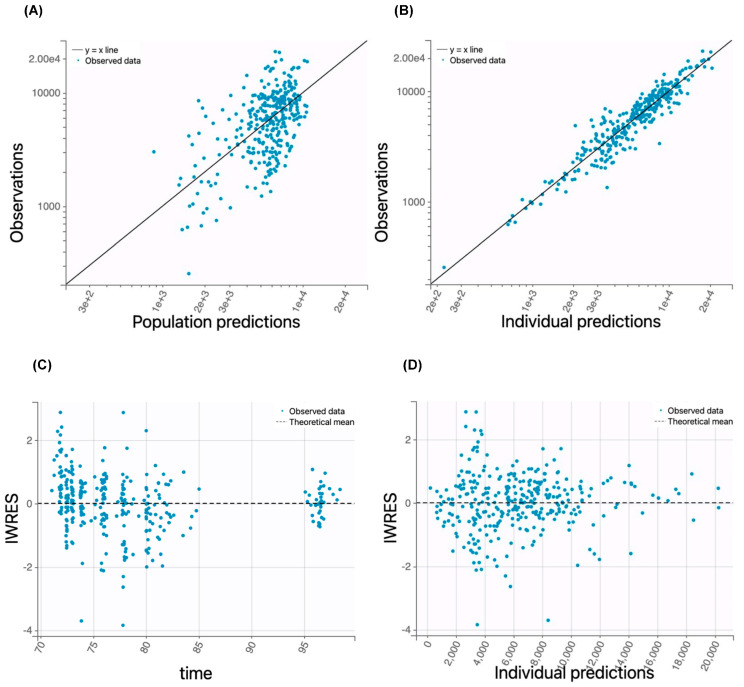
Goodness-of-fit plots for the total plasma pamiparib concentration–time course data fitted by the final covariate model. (**A**) The relationship between the observed and population parameter-predicted total drug concentrations. The solid line is the line of identity. (**B**) The relationship between the observed and individual parameter-predicted total drug concentrations. The solid line is the line of identity. (**C**) Individual weighted residues (IWRESs) versus time. The dashed line is the mean IWRES. (**D**) Individual weighted residues (IWRESs) versus the individual parameter-predicted total drug concentrations. The dashed line is the mean IWRES.

**Figure 2:**
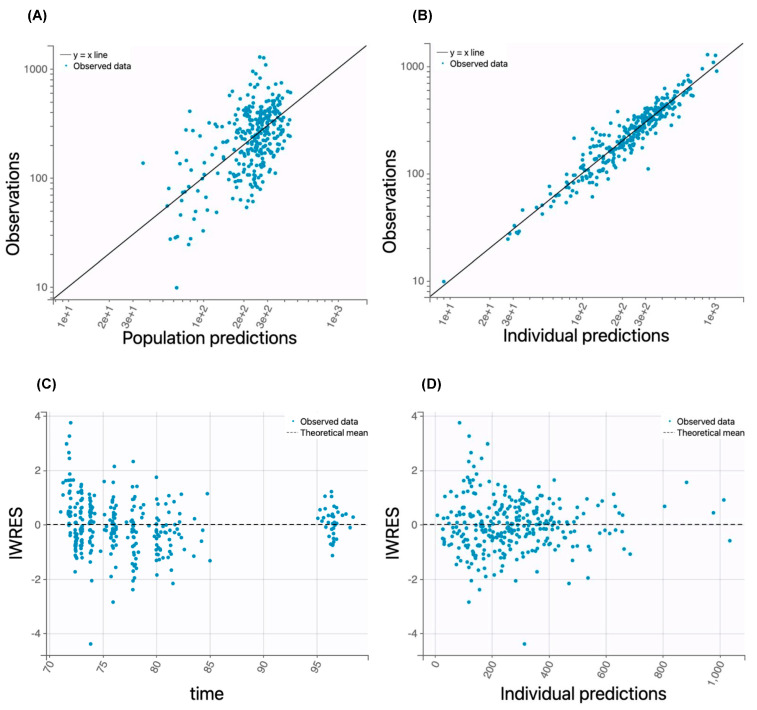
Goodness-of-fit plots for the unbound plasma pamiparib concentration–time course data fitted by the final covariate model. (**A**) The relationship between the observed and population parameter-predicted unbound drug concentrations. The solid line is the line of identity. (**B**) The relationship between the observed and individual parameter-predicted unbound drug concentrations. The solid line is the line of identity. (**C**) Individual weighted residues (IWRESs) versus time. The dashed line is the mean IWRES. (**D**) Individual weighted residues (IWRESs) versus the individual parameter-predicted unbound drug concentrations. The dashed line is the mean IWRES.

The prediction performance of the final population PK model was evaluated using goodness-of-fit plots, nonparametric bootstrap analysis, visual predictive check, and prediction distribution plots. The goodness-of-fit plots (Figure 1 and Figure 2) demonstrated a good correlation between the model-predicted total or unbound pamiparib concentrations and the observed data, and the residual plots showed randomly scattered residuals around the horizontal zero line. These data collectively indicated no systematic prediction bias.

The robustness of the parameter estimates was assessed via bootstrap analysis. Table 3 summarizes the population mean PK parameter estimates of the final model alongside the mean and 95% confidence intervals of the bootstrap-derived estimates. The agreement between the population mean PK parameter estimates and the mean of bootstrap replicates, with both falling within the 95% confidence intervals of bootstrap replicates, confirmed the stability of parameter estimation from the final population PK model.

**Figure 3:**
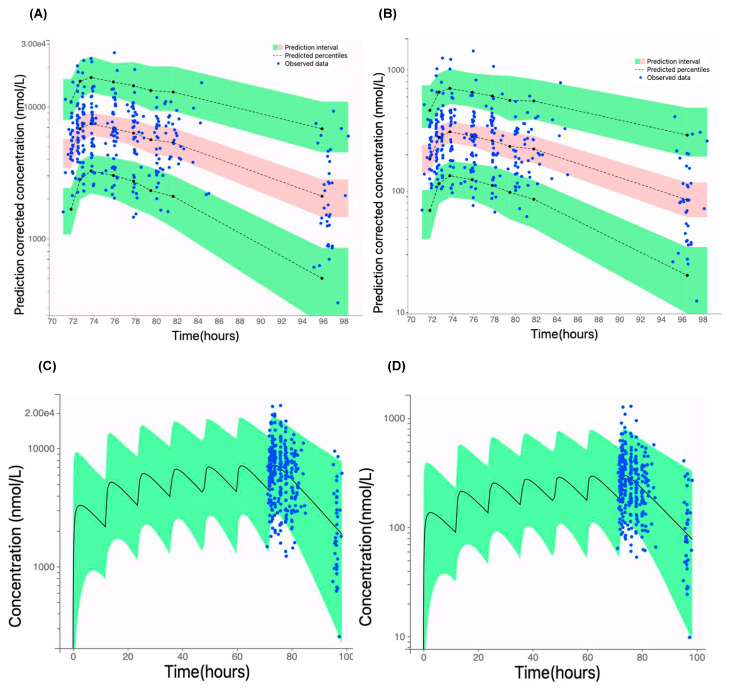
Model evaluation. (**A**,**B**) Visual predictive check of the final covariate model for the total and unbound pamiparib plasma concentrations. The dashed lines represent the 50th, 5th, and 95th percentiles of the model-predicted concentration–time profiles. The shaded areas represent the 95% confidence intervals of the percentiles. Symbols represent the observed drug concentration data. (**C**,**D**) Prediction distribution plots for the total and unbound pamiparib plasma concentration–time profiles following multiple-dose oral administration (60 mg BID). The solid lines represent the model-predicted population mean drug concentration–time profile, and the shaded areas represent the 95% confidence intervals. Dot symbols represent observed concentration data.

Model predictive performance was evaluated through visual predictive checks (Figure 3A,B). The observed total or unbound pamiparib concentrations fell within the 95% prediction interval and aligned well with the simulated percentiles (50th, 5th, and 95th percentiles), demonstrating that the model adequately captured both the central trend and variability of the observed data. Prediction distribution plots (Figure 3C,D) further assessed predictive accuracy, detecting potential systematic deviations due to model misspecification, overdispersion, or unexplained variability. As shown in Figure 3C,D, >95% of the observed data fell within the 95% prediction intervals and aligned well with the simulated percentiles, indicating no model misspecification.

Collectively, the goodness-of-fit plots, bootstrap analysis, visual predictive checks, and prediction distribution plots provided a comprehensive assessment of parameter stability and model predictive performance. These results confirmed that the developed one-compartment population PK model with a combined residual error model adequately well characterized the observed total and unbound pamiparib plasma concentration–time profiles in glioblastoma patients.

### 3.3. Model Simulation

**Figure 4:**
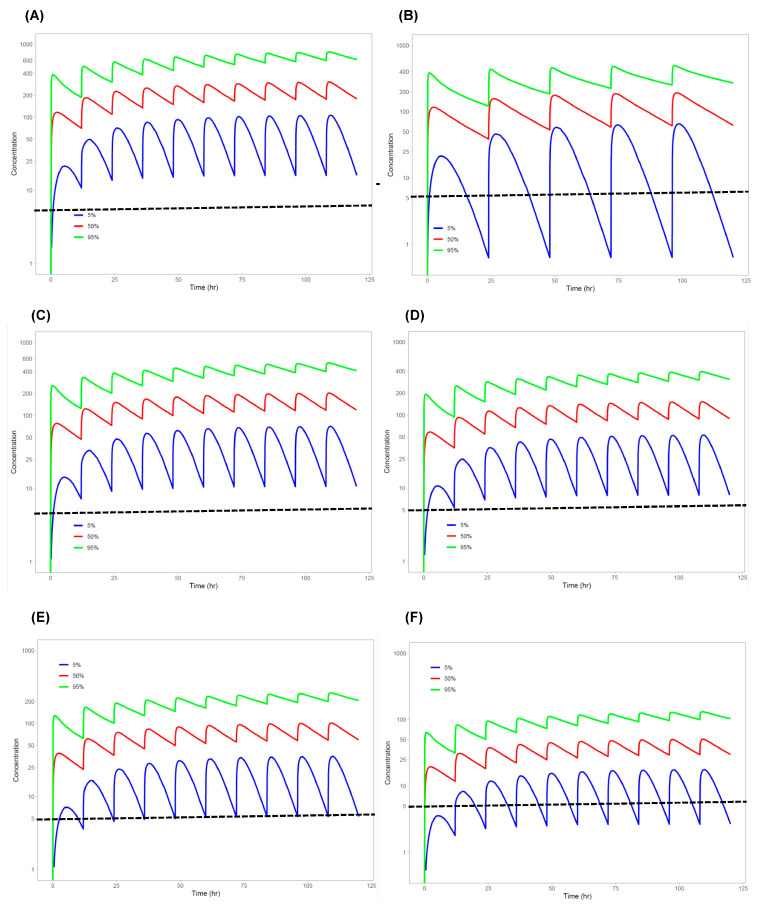
Model-predicted unbound pamiparib concentration–time profiles following oral administration of pamiparib at different dosing regimens: (**A**) 60 mg BID, (**B**) 60 mg QD, (**C**) 40 mg BID, (**D**) 30 mg BID, (**E**) 20 mg BID, and (**F**) 10 mg BID. The red, green, and blue lines represent the 50th, 5th, and 95th percentiles of the predicted concentration profiles. The dashed lines represent the target engagement ratio of 5, which is assumed to be the threshold for therapeutic efficacy.

Figure 4 shows the population PK model-predicted unbound pamiparib plasma concentration–time profiles (including the 50th, 5th, and 95th percentiles) following 5 days of twice-daily oral administration at doses of 10, 20, 30, 40, and 60 mg in a general patient population. Table 4 summarizes the model-predicted steady-state peak, trough, and average plasma concentrations of the unbound pamiparib following twice-daily oral doses of 10–60 mg. When doses increased from 10 to 60 mg BID, the median trough steady-state unbound drug concentrations (C_ss,min_) ranged from 29 to 175 nM, with the 5th percentile ranging from 3 to 16 nM and the 95th percentile ranging from 97 to 581 nM. Given pamiparib’s potent PARP inhibition (IC50, ~1 nM) and strong PARP trapping ability, a target engagement ratio (i.e., unbound C_ss,min_/IC50) of 5 or above was considered sufficient for full target engagement and pharmacological activity. Model predictions suggested that doses as low as 20 mg BID may be adequate for therapeutic effects, even in patients with rapid clearance, where the unbound pamiparib C_ss,min_ was approximately 5 nM (i.e., 5th percentile) (Table 4).

## 4. Discussion

Due to its strong PARP–DNA trapping ability, favorable PK properties, CNS penetration, and therapeutic potential, pamiparib was evaluated, as a monotherapy or in combination with other therapies, for the treatment of various cancers, particularly BRCA-mutated or homologous recombination-deficient tumors [1,3,4,5,6,18]. In this study, we developed a population PK model that well characterized the plasma PK profiles of both total and unbound pamiparib in glioblastoma patients. The model provides a robust framework for optimizing dosing regimens to enhance the clinical development and therapeutic success of this novel PARP inhibitor in oncology.

The pamiparib plasma pharmacokinetic profile following oral administration is characterized by rapid absorption (population mean Ka, 1.58 h^−1^), low apparent clearance (population mean CL/F, 2.59 L/h), which is significantly lower than human hepatic blood flow (~ 80 L/h), extensive distribution (population mean V/F, 44 L), and high plasma protein binding (population mean Fu, 0.041). These PK parameters align well with pamiparib’s physicochemical properties and metabolic characteristics. Pamiparib is a small-molecule drug (molecular weight: 298.3) with moderate lipophilicity (LogP: 2.03), enabling good passive permeability across biological membranes. Notably, it is not a substrate for the efflux transporters ABCB1 and ABCG2, which may contribute to its extensive tissue distribution, including good penetration into the brain [1,9]. Consistent with its low intrinsic clearance determined in human liver microsomes, a mass balance study in patients with solid tumors demonstrated that unchanged pamiparib is the primary circulating component in plasma, comprising 67% of total radioactivity, indicating limited metabolic conversion in humans [18]. Collectively, these observations, in line with our estimated population PK parameters, support that pamiparib exhibits high oral bioavailability (driven by efficient passive permeability and an insignificant first-pass effect), low systemic clearance (due to limited liver metabolism), and extensive tissue distribution.

The population PK analysis suggests that pamiparib exhibits a large interindividual PK variability in patients, with > 50% IIV observed in Ka, V/F, and CL/F (Table 2). While creatinine clearance and age were identified as statistically significant covariates influencing V/F and CL/F for both total and unbound drug, they account for only about 22% and 5% of the respective IIV (Table 2). This suggests that other unassessed patient factors contribute substantially to the interindividual PK variability of pamiparib. One potential factor is the activity of liver enzymes, such as CYP3A4 and CYP2C8, which play key roles in pamiparib metabolism [18]. Potential drug–drug interactions should be considered, particularly with strong CYP3A4 inhibitors or inducers, although the present population PK analysis did not identify any concomitant drugs as significant covariates on pamiparib PK. A mass balance study in patients revealed that pamiparib metabolites contribute to approximately 33% of the total radioactivity in plasma [18]. The major metabolic pathways involve the oxidation and, to a lesser extent, phase II conjugation of oxidative metabolites. M3 (oxy-dehydro pamiparib) is the most abundant metabolite in human plasma, urine, and feces [18]. Pamiparib and its metabolites undergo both renal and fecal excretion, accounting for approximately 58% and 27% of total radioactivity, respectively [18]. The present study enrolled patients with adequate liver and renal functions. However, given the critical roles of liver metabolism and renal excretion in pamiparib disposition, the impact of severe liver impairment or severe renal dysfunction (creatinine clearance < 30 mL/min) on pamiparib PK remains to be investigated.

Pamiparib is a potent and selective inhibitor of PARP1 and PARP2 enzymes, with a half-maximal inhibitory concentration (IC50) of approximately 1 nM [2]. In addition, it exhibits strong PARP trapping potency, surpassing olaparib and rucaparib but slightly weaker than niraparib and much weaker than talazoparib (the most potent PARP trapper, ~100-fold stronger than olaparib) [19]. PARP trapping is a key mechanism underlying the cytotoxicity of PARP inhibitors, particularly in BRCA-mutated and homologous recombination-deficient cancers [20,21]. Trapped PARP-DNA complexes were more cytotoxic than unrepaired single-strand breaks caused by PARP inactivation, positioning PARP inhibitors as agents that not only inhibit enzyme activity but also act as poisons by trapping PARP on damaged DNA [20,21].

Pamiparib monotherapy has demonstrated clinically meaningful antitumor activity in patients with platinum-sensitive ovarian cancer harboring germline BRCA mutations [3]. However, its efficacy is limited in patients with recurrent ovarian cancer previously treated with PARP inhibitors [22]. In general, PARP inhibitor monotherapy (e.g., olaparib, rucaparib, niraparib, and pamiparib) is most therapeutically effective in maintenance settings for tumors (e.g., breast and ovarian cancer) with BRCA mutations or homologous recombination deficiency [23]. However, broader patient populations or aggressive tumors often require combination therapy for improved clinical efficacy [24]. Combining a PARP inhibitor and a chemotherapeutic agent can lead to synergistic effects, as PARP inhibition prevents DNA repair following DNA damage caused by chemotherapy (e.g., platinum-based drugs and alkylating agents) [24,25]. Additionally, combination strategies may help overcome resistance mechanisms that can develop with monotherapy [24,25]. The efficacy and toxicity of pamiparib-based combination therapies with DNA-damaging agents, radiation, targeted therapies, and immunotherapy are under clinical investigation across multiple cancer types [7]. While pamiparib monotherapy has a manageable safety profile, with primarily hematologic toxicities such as anemia and neutropenia, combination regimens often increase the risk of hematologic and gastrointestinal toxicities. Therefore, optimizing pamiparib dosage or scheduling in combination therapies is critical for balancing efficacy and tolerability to maximize clinical benefit.

The dose– or exposure–response relationships for PARP inhibitors have been explored in preclinical and clinical studies [26,27]. In general, these drugs exhibit a plateau effect, meaning that increasing the drug dose beyond a certain threshold does not significantly enhance efficacy but does increase toxicity [26]. For instance, rucaparib has a threshold dose where higher concentrations do not improve efficacy but increase toxicity; olaparib shows a nonlinear dose–response relationship; and niraparib exhibits dose-dependent hematologic toxicity [26]. Additionally, an imaging study of olaparib in SCLC PDX models demonstrated that olaparib achieved 50% target engagement at the dose of 3.17 mg/kg (ED50) and complete target engagement at 15 mg/kg (~ 5-fold of ED50), with no further benefit beyond 15 mg/kg [27]. These findings align with the observed plateau effect in clinical settings. While the exact dose– or exposure–response relationship for pamiparib is yet to be fully established, a similar plateau effect is expected.

The current standard pamiparib dose (60 mg BID), established as the maximum tolerated dose in monotherapy [4], may exceed the optimal biological dose required for therapeutic efficacy while minimizing toxicity. Given pamiparib’s excellent tissue distribution (including brain penetration), its unbound plasma concentrations could serve as a surrogate for pharmacologically active concentrations at the target site. The target engagement ratio, defined as the unbound drug trough steady-state plasma concentration (C_ss,min_) relative to the IC50 for PARP inhibition (1 nM), can be used as an indicator of potential therapeutic effects. Model simulations following twice-daily oral administration at doses of 10, 20, 30, 40, and 60 mg suggest that pamiparib achieves the target engagement ratio (C_ss,min_/IC50) of 29–175 (50th percentile) in patients with a typical population mean apparent clearance (CL/F), 97–581 (95th percentile) in patients with low CL/F, and 3–16 (5th percentile) in patients with high CL/F (Figure 4 and Table 4). Assuming that a target engagement ratio of 5 or above is sufficient for full target engagement and pharmacological activity, doses as low as 20 mg BID may be adequate for therapeutic effects even in patients with rapid clearance, where the target engagement ratio is 5 (i.e., 5th percentile) (Figure 4 and Table 4). These findings support further clinical investigation into dose reduction to optimize pamiparib’s benefit-to-risk ratio, particularly in maintenance settings for BRCA-mutated or homologous recombination-deficient tumors, which may respond to lower PARP inhibitor doses. Furthermore, in combination therapies, where dose–response dynamics may shift due to synergistic effects, lower pamiparib doses would enhance tolerability while maintaining efficacy. Continued clinical investigation into the optimization of the pamiparib dosing regimen is essential for refining its clinical application and maximizing therapeutic benefit.

In conclusion, the total and unbound pamiparib plasma PK are well characterized by a linear one-compartment model, with creatinine clearance as the significant covariate on V/F. Model simulations support further clinical investigation into dose reduction to optimize the benefit-to-risk ratio of pamiparib, particularly in combination therapies.

## Figures and Tables

**Table 1:** Patient demographic and characteristic data.

	Median (Range) or Number of Patients
**Baseline characteristics**	
Race (white/non-white) ^†^	38/3
Sex (male/female) ^†^	20/21
Age (years) *	60 (31–80)
Weight (kg) *	80 (45–129)
Height (cm) *	173 (155–193)
BSA ($m^{2}$) *	1.99 (1.41–2.53)
**Liver function** *	
Predose total bilirubin (mg/dL)	0.5 (0.3–1.8)
Post-operation total bilirubin (mg/dL)	0.5 (0.2–1.5)
Predose AST (IU/L)	19 (9–44)
Post-operation AST (IU/L)	23 (9–106)
Predose ALT (IU/L)	24 (8–135)
Post-operation ALT (IU/L)	45 (14–193)
Predose plasma albumin (mg/dL)	4.1 (3.5–5)
Post-operation plasma albumin (mg/dL)	3.9 (3–4.5)
**Kidney function** *	
Predose serum creatine (mg/dL)	0.83 (0.56–1.34)
Post-operation serum creatine (mg/dL)	0.75 (0.41–1.3)
Predose creatine clearance (mL/min)	98 (39–154)
Post-operation creatine clearance (mL/min)	111 (59–169)
Predose GFR (mL/min)	92 (48–116)
Post-operation GFR (mL/min)	101 (48–117)
**Concomitant drugs**	
Coadministered drugs during the trial (yes/no) ^†^	36/5
Dexamethasone (given/not given) ^†^	34/7
Total dexamethason dose (mg) *	19 (0–66)

* Values are shown as median (range). ^†^ Data indicate the number of patients. Abbreviations: AST, aspartate aminotransferase; ALT, alanine aminotransferase; GFR, glomerular filtration rate.

**Table 2:** Population PK parameters for the total and unbound pamiparib estimated from the base (structure) model and final covariate model.

Parameter *	Total Pamiparib		Unbound Pamiparib	
	Base Model	Final Model	Base Model	Final Model
OFV	9226	9210	9226	9212
TV_KA (h^−1^)	1.64	1.58	1.55	1.7
TV_V/F (L)	44	44 ^†^	1017	1060 ^†^
TV_CL/F (L/h)	2.73	2.59 ^‡^	65.0	62.5 ^‡^
TV_Fu	0.042	0.041	0.042	0.042
$\theta_{1}$ (Ka)	1.64 (42)	1.58 (42)	1.55 (51)	1.7 (44)
$\theta_{2}$ (V/F)	44 (9.7)	15 (28)	1017 (9.1)	402 (28)
$\theta_{3}$ (CL/F)	2.73 (8.0)	6.76 (38)	65.0 (8.2)	163 (39)
$\theta_{4}$ (Fu)	0.042(2.8)	0.041 (2.8)	0.042 (2.7)	0.042 (2.6)
$\beta_{1}$ (PCC on V/F)	-	0.0094 (25)	-	0.0087(26)
$\beta_{2}$ (Age on CL/F)	-	−0.016 (37)	-	−0.016 (38)
IIV of Ka (%)	397 (18)	410 (20)	612 (21)	380 (20)
IIV of V/F (%)	53 (16)	41 (17)	50 (17)	41 (19)
IIV of CL/F (%)	53 (11)	50 (11)	54 (11)	52(11)
IIV of Fu (%)	12 (22)	12 (20)	112 (24)	11 (28)

* PK parameter estimates are expressed as the population mean (% relative standard error of estimation). ^†^ $TV\_V=\theta_{2}*e^{\beta_{1}*PCC}$, with the population median PCC being 111.5. ^‡^ $TV\_CL=\theta_{3}*e^{\beta_{2}*Age}$, with the population median age being 60. Abbreviations: θ, population mean PK parameter estimate from the final covariate model; TV, typical population mean PK parameter; Ka, elimination rate constant; CL/F, apparent volume of distribution; V/F, apparent volume of distribution; Fu, fraction of unbound drug in plasma; PCC, post-operation creatine clearance; IIV, interindividual variability.

**Table 3:** Population PK parameters estimated from the final covariate model and bootstrap analysis.

	Total Pamiparib			Unbound Pamiparib		
Parameter	Population Mean	Bootstrap Mean	Bootstrap 95% CI	Population Mean	Bootstrap Mean	Bootstrap 95% CI
$\theta_{1}$ (Ka) (h^−1^)	1.58	1.71	(0.64, 4.15)	1.7	1.86	(0.67, 4.07)
$\theta_{2}$ (V/F) (L)	15	17	(8, 30)	402	441	(219, 787)
$\theta_{3}$ (CL/F) (L/h)	6.76	7.76	(3.6, 15.9)	163	185	(83, 454)
$\theta_{4}$ (Fu)	0.041	0.041	(0.039, 0.044)	0.042	0.041	(0.039, 0.044)
$\beta_{1}$ (PCC)	0.0094	0.0089	(0.0035, 0.015)	0.0087	0.0083	(0.0027, 0.014)
$\beta_{2}$ (Age)	−0.016	−0.017	(−0.03, −0.0054)	−0.016	−0.016	(−0.032, −0.0053)
Ka_SD	1.7	1.6	(1.0, 2.3)	1.66	1.66	(1.1, 2.34)
V/F_SD	0.39	0.35	(0.16, 0.5)	0.39	0.35	(0.16, 0.5)
CL/F_SD	0.47	0.46	(0.35, 0.56)	0.49	0.46	(0.37, 0.37)
Fu_SD	0.12	0.11	(0.06, 0.16)	0.11	0.11	(0.059, 0.15)

Abbreviations: θ, population mean PK parameter estimate; Ka, elimination rate constant; CL/F, apparent volume of distribution; V/F, apparent volume of distribution; Fu, fraction of unbound drug in plasma; PCC, post-operation creatine clearance; SD, standard deviation.

**Table 4:** Population PK model-predicted steady-state plasma concentrations of the unbound pamiparib at the 5th, 50th, and 95th percentiles in a general patient population following 5 days of treatment at different dosing regimens.

Dosing Regimen		C_ss,max_			C_ss,min_			C_ss,ave_	
	5th	50th	95th	5th	50th	95th	5th	50th	95th
60 mg BID	105	302	774	16	175	581	47	233	673
60 mg QD	63	193	485	0.6	61	253	14	115	357
40 mg BID	68	201	515	11	117	388	31	155	448
30 mg BID	53	151	387	8	88	291	24	116	336
20 mg BID	35	101	258	5	58	194	16	78	224
10 mg BID	18	50	129	3	29	97	8	39	112

Abbreviations: C_ss,max_, steady-state peak concentration; C_ss,min_, steady-state trough concentration; C_ss,ave_, average steady-state drug concentration; BID, twice-daily dosing; QD, once-daily dosing.

## Data Availability

The data generated in this study are available within the paper. The raw data used for the population PK analysis and creating graphs are available from the corresponding author upon reasonable request.
